# Advancing Pediatric Cognitive Health: Psychometric Evaluation and IRT- and Regression-Based Norms for Two Neuropsychological Measures in Colombian Children and Adolescents

**DOI:** 10.3390/healthcare13212683

**Published:** 2025-10-23

**Authors:** Eliana María Fuentes Mendoza, Laiene Olabarrieta-Landa, Clara Sancho-Domingo, Oscar Teijido, Juan Carlos Arango-Lasprilla, Diego Rivera

**Affiliations:** 1 Department of Health Science, Public University of Navarre, 31006 Pamplona, Spain; fuentes.151056@e.unavarra.es (E.M.F.M.); laiene.olabarrieta@unavarra.es (L.O.-L.); oscar.teijido@unavarra.es (O.T.); diego.rivera@unavarra.es (D.R.); 2 Center for Experimentation and Applied Research in Psychometrics and Evaluation (CEI-TEST), National Autonomous University of Honduras, Boulevard Suyapa, Tegucigalpa 11101, Honduras; 3 Instituto de Investigación Sanitaria de Navarra (IdiSNA), 31008 Pamplona, Spain; 4 Department of Cell Biology and Histology, University of the Basque Country UPV/EHU, 48940 Leioa, Spain; 5 IKERBASQUE, Basque Foundation for Science, 48009 Bilbao, Spain

**Keywords:** normative data, Colombian children, item response theory, regression models, Rey–Osterrieth Complex Figure, Shortened Version of the Token Test

## Abstract

**Objective:** To evaluate the psychometric properties of the short version of the Token Test (SVTT) and the Rey–Osterrieth Complex Figure (ROCF) using an item response theory (IRT) framework and to establish normative data for Colombian children and adolescents based on ability scores. **Methods:** A total of 668 healthy participants aged 6–17 years took part in this study. Factorial structure was assessed through confirmatory factor analysis (CFA). Item parameters were estimated using a two-parameter logistic (2PL) model for the SVTT, which accounts for both item difficulty and discrimination in dichotomous responses, and a graded response model (GRM) for the ROCF, suitable for items scored on ordered categories reflecting increasing levels of performance accuracy and Differential Item Functioning (DIF) analysis was conducted to assess potential bias related to sex. Reliability was examined using the Test Information Function (TIF), internal consistency throughout Cronbach’s alpha, and the influence of sociodemographic variables was analyzed through regression models. **Results:** CFA confirmed unidimensionality for all measures. For most items, moderate-to-low ability was sufficient to achieve the highest scores in the ROCF, and low ability in the SVTT. DIF analysis indicated no meaningful sex-related bias in any of the subtests. Both tests showed excellent reliability and internal consistency. Copy scores were influenced by polynomial age and parents’ mean years of education (MPE), while both immediate recall in the ROCF and SVTT were affected by MPE and the interaction of logarithmic age. **Conclusions:** This study provides strong psychometric evidence and, together with the integration of digital tools for generating normative data, represents a meaningful advancement in neuropsychological assessment.

## 1. Introduction

Neuropsychological assessment is a comprehensive, structured process for evaluating cognitive and psychological functioning, which encompasses data from multiple sources, including standardized tests, to support clinical decision making. These tests allow clinicians and researchers to compare patients’ performance across diverse populations [1]. Due to their central role, their utility depends on robust psychometric properties, a requisite for accurate assessments, diagnosis and clinical decision making [2].

Neuropsychological tests are intended to be reliable, valid, and adapted to the cultural, linguistic, and demographic contexts of the population to ensure representativeness and minimize measurement bias [2,3]. Instruments developed, validated and normed in different cultural and linguistic settings may fail to capture cognitive performance in other populations, leading to misdiagnosis and limited generalizability of findings [3,4,5]. Despite this, validation and normative studies in diverse populations remain insufficient, and it is still necessary to validate commonly used cognitive function tools for clinical practice and research.

The Rey–Osterrieth Complex Figure [6] and the Token Test [7] are two of the most used neuropsychological measures that were developed and normed on relatively homogeneous samples, lacking adequate representation of specific populations [5]. The ROCF assesses the visuo-constructional ability and non-verbal memory, including copying and recall tests, and has been used for brain injury or cognitive disorders and the general population with adequate reliability and validity [8,9]. On the other hand, TT evaluates verbal comprehension through the execution of spoken commands and serves as a diagnostic tool for certain language disorders, such as aphasia or dementia [10,11]. The TT has demonstrated good reliability, validity, and adequate classification power [10,12]. More efficient adaptations of the TT have been developed, such as the Shortened Version of the Token Test [13], which has also shown adequate psychometric properties for assessing language difficulties in both adults and children [11,14].

However, most neuropsychological tests have been developed and validated within the framework of Classical Test Theory (CTT), including ROCF and TT [5]. The CTT approach estimates an individual’s observed score as the sum of their true ability and measurement error and provides metrics of reliability and validity that are useful for both clinical and research purposes [15]. However, CTT presents some limitations that include relying on test-level statistics, assuming equal item contribution, and being sample-dependent. These constraints can limit the interpretability, precision, and generalizability of scores, particularly when applied across diverse cultural or linguistic groups [15].

In contrast, Item Response Theory (IRT) emerged as an alternative to address some of the CTT limitations [16,17]. The IRT focuses on item-level analysis, modeling the probability of a given response as a function of an individual’s latent ability (*θ*), and specific item parameters, such as difficulty and discrimination, and how it distinguishes between individuals with different ability levels [17]. This approach provides a more accurate estimation of individuals’ ability levels in specific cognitive functioning, regardless of individuals’ differences. Also, IRT incorporates Differential Item Functioning (DIF) to detect and reduce measurement bias across demographic groups [17] and it has gained popularity in neuropsychological assessment due to the limitations of CTT, which relies on total scores and fails to capture the information provided by individual items. Prior studies have already provided empirical support for the superior performance of IRT-based scoring compared with CTT approaches. Specifically, IRT models have been shown to produce lower Type I error rates, greater statistical power, and more accurate effect-size estimation [18,19], as well as higher sensitivity to true individual change, as reflected by the Magnitude of True Change index [20].

In the ROCF, the total score is commonly used to determine an individual’s performance in copy or memory conditions. However, this limits the interpretation of item-level performance. Two individuals may obtain the same total score but differ on specific items, reflecting different underlying cognitive difficulties [15]. IRT allows us to identify which of the ROCF 18 scoring units are most difficult or discriminative, and therefore, estimate a more precise and person-specific ability score. A similar limitation exists for TT, where clinicians often rely solely on the total score. Moreover, this test was originally designed with increasing items of difficulty, making IRT ideal for evaluating whether the original intended item order remains for a particular population.

IRT has gained increasing attention in neuropsychological research for its ability to identify redundant items and effective items in distinguishing abilities, optimizing test efficiency and improving interpretation guidelines in neuropsychological assessment [8,17,21,22]. Despite its advantages, IRT-based research remains limited, especially in low- and middle-income countries. Few IRT studies focused on pediatric populations in Latin America, thereby creating a major gap in culturally appropriate evidence [23].

Although normative data available for the ROCF and the SVTT exist for various populations, including Spanish-speaking adults and pediatric populations from Spain and several Latin American countries [11,14], most are based on the CTT framework and lack of psychometric analyses of the neuropsychological test. Norms based on raw scores under the CTT may therefore lack the precision required for sensitive clinical decision making [15]. Therefore, it is necessary to conduct validation studies based on IRT that also provide normative data to determine whether an individual’s performance within a specific population is ‘normative’ or indicative of impairments [1].

Improving measurement precision is critical for the early detection of cognitive impairment and for preventing developmental decline, including behavioral difficulties and declines in attention, language, and cognitive performance, even among initially high-functioning children [24]. In this regard, there is a need for valid and culturally sensitive appropriate tools to detect and monitor cognitive changes over time, particularly during childhood, when declines may go unnoticed, reducing the chances for early intervention.

Based on previous scientific literature, there is a growing need to improve the use and adaptation of neuropsychological instruments, particularly those applied across different cultural and linguistic contexts. The inappropriate use of these instruments may compromise the validity of clinical practice and increase the risk of biased interpretations [3,4,5], especially when transporting tests and norms from other cultural-linguistic contexts to Spanish-speaking populations, where validation studies are still scarce [23]. In addition, norms based solely on the CTT framework provide limited assessment precision [15] and may not be sufficiently sensitive to guide decision making in pediatric settings, where applications of IRT remain uncommon [23]. In this regard, children and adolescents require assessment tools that can deliver individualized evaluations and accurately differentiate among varying levels of ability, enabling clinicians to make better-informed and fairer diagnostic judgments [17].

To fill this gap, the present study provides IRT-based psychometric analyses and regression-adjusted normative data for two widely used neuropsychological tests (ROCF and SVTT) in a pediatric, Spanish-speaking, Latin American population. The aims of this study were: (1) to examine the psychometric properties of the ROCF and SVTT, including validity, reliability, item difficulty and discrimination parameters, and to evaluate Differential Item Functioning [DIF] by sex; and (2) to generate normative data for Colombian children using IRT and regression models. This study aims to enhance the use of neuropsychological assessment tools in specific populations and to minimize the risk of bias in clinical practice among pediatric patients.

## 2. Materials and Methods

### 2.1. Participants

The initial sample included 690 children and adolescents from four Colombian cities Medellín, Cali, Ibagué and Bogotá. To be included in the study, participants had to meet the following criteria: (a) be between 6 and 17 years old, (b) be born and residing in Colombia, (c) have an IQ ≥ 80 on the Test of Nonverbal Intelligence [25], and (d) score < 19 on the Children’s Depression Inventory [26]. Exclusion criteria included: (a) reported learning difficulties, (b) a history of psychoactive substance use (for adolescents), and (c) a score < 5 on the Alcohol Use Disorder Identification Test (AUDIT-C) [27]. Verification of inclusion and exclusion criteria was conducted through an interview with the participants’ parents or caregivers. Due to incomplete demographic data, 22 participants were excluded, resulting in a final sample of 668 individuals. Of the total participants, 54.19% were female (n = 374). The average age was 11.34 years (SD = 3.28), and the Mean Years of Parental Education (MPE) was 12.15 years (SD = 3.62). Half of the sample attended public schools, while the other half attended private schools. The distribution across cities were: Medellín (n = 181), Cali (n = 141), Ibagué (n = 190), and Bogotá (n = 156).

### 2.2. Instruments

#### 2.2.1. Rey-Osterrieth Complex Figure (ROCF)

The ROCF assesses visual–spatial construction and immediate visual memory. In this study, the A-version of the figure, which composed of 18 elements was used. The test is administered in two phases: first, the participant is asked to copy the figure; then, after a delay of three minutes, the figure is removed and the participant is instructed to reproduce it from memory. Scoring is based on the accuracy and placement of each element: 2 points are awarded for accurate and correctly placed reproductions, 1 point for elements that are distorted or incomplete but correctly located, or for complete elements that are poorly positioned. If the element is both distorted or incomplete and misplaced, 0.5 points are assigned. A score of 0 is given when the element is omitted or unrecognizable [6].

#### 2.2.2. Shortened Version of the Token Test (SVTT)

The SVTT is designed to measure verbal comprehension through the execution of verbal commands involving tokens of varying shapes, sizes, and colors. The task consists of six parts (36 items) with increasing complexity, where participants are instructed to manipulate 20 tokens according to specific verbal directions. Each correct response receives 1 point; if the correct response is given only after repeating the instruction, 0.5 points are awarded [13]. However, in line with this study’s criteria, all 0.5-point responses were recoded as 0. No points are awarded if the participant fails to respond correctly, even after the instruction is repeated.

### 2.3. Procedure

This study is part of a larger multicenter project aimed at generating normative data for Spanish-speaking populations. Participants completed a battery of neuropsychological tests, including the ROCF and SVTT, which were the only tests suitable for item-level analysis. To ensure consistency and minimize potential bias, the order of test administration was randomized for all participants. Additionally, a Microsoft Excel template was used to minimize data entry errors. The study was approved by the Ethics Committee of the University of the North and Pedagogical and Technological University of Colombia and conducted in accordance with the ethical principles of the Declaration of Helsinki. Parents or legal guardians who agreed to participate signed an informed consent form and completed a questionnaire gathering sociodemographic data, medical history, and the child’s health status. All assessments were conducted individually at participating schools. Data collection took place between July and December 2017. Participation was entirely voluntary, and no monetary compensation was offered.

### 2.4. Statistical Analysis

#### 2.4.1. Psychometric Properties

To assess the construct validity of each instrument, we conducted separate Confirmatory Factor Analyses (CFAs) using the Diagonally Weighted Least Squares (DWLS) estimator, which is well-suited for ordinal data. These analyses aimed to evaluate whether the items from each test reflected a single underlying factor, that is, whether the assumption of unidimensionality was supported. CFAs model fit was evaluated using several standard indices like the chi-square to degrees of freedom ratio $x^{2}/df$, where values of ≤3 indicate adequate fit [28], Comparative Fit Index (CFI) and Tucker–Lewis Index (TLI), for which values ≥ 0.95 reflect excellent fit; and the Root Mean Square Error of Approximation (RMSEA), considered optimal when ≤0.06, accompanied by its 90% confidence interval. The Standardized Root Mean Square Residual (SRMR), with values < 0.08, was also used to determine the acceptability of the models [29].

To further investigate item functioning, IRT models were employed to estimate item-level parameters. Dichotomous SVTT items were modeled using a two-parameter logistic (2PL) model, which allows both item difficulty (b) and discrimination (a) to vary across items, offering greater flexibility than the Rasch model [30]. In contrast, the ROCF copy and immediate recall tasks, which consist of polytomous items, were analyzed using the Graded Response Model (GRM). This model estimates the probability of responding in each category as a function of the latent trait level (*θ*) and evaluates how well each item discriminates across different levels of the trait [31].

The estimated ability parameter (*θ*) obtained from the IRT models was used as a refined measure of individual performance. This parameter represents each person’s standing on the underlying trait (e.g., visuoconstructive ability or verbal comprehension) and is derived by modeling their pattern of responses in relation to the psychometric characteristics of each item (such as how difficult or discriminative the items are). Unlike raw scores, *θ*-score provides a standardized, interpretable metric that enables more accurate comparisons across individuals and across different sets of items. Differential Item Functioning (DIF) was analyzed to identify potential bias across specific groups, focusing on sex as the variable of interest [32]. Logistic regression was employed for DIF detection, leveraging trait scores derived from IRT, and this method iteratively adjusts item parameters based on group differences (in this case, boys and girls), as modeled through a series of regressions and evaluates the magnitude of explained variance through the incremental ΔR^2^ [33].

Measurement precision (reliability) was evaluated for both the ROCF and SVTT using IRT methods. Test information functions (TIF) were computed to assess the precision of measurement across ability score (*θ*), with higher information values $I\left(\theta \right)$ indicating greater reliability at specific trait levels. The relationship between information and measurement error is given by $I\left(\theta \right)=\;1/{\left[SEM(\theta )\right]}^{2}$, where SEM is the standard error of measurement.

#### 2.4.2. The Effects of Age, Gender, and MPE on the *θ*-Scores

To explore the influence of demographic variables on test performance, multiple linear regression analyses were conducted using the ability estimates (*θ*) as dependent variables for ROCF copy, ROCF immediate recall, and SVTT. To examine the predictive value of age, MPE, and sex, we compared two families of models differing in the transformation applied to the age variable: one included a logarithmic transformation *ln*(Age), and the other a quadratic orthogonal polynomial effect (Age and Age^2^). The quadratic formulation was motivated by the previous literature reporting nonlinear age effects in neuropsychological tasks [34,35], and was expressed $\theta_{i}=\beta_{0}+\;\beta_{1}{\left(Age\right)}_{i}+\beta_{2}\;{\left(Age\right)}_{i}^{2}+\;\beta_{3\;}\left[{MPE}_{i}\right]+\;\beta_{4}\;{\left(Sex\right)}_{i}+\beta_{k}\;{Interactions}_{i}+\varepsilon_{i}$.

However, from a clinical perspective, a monotonic increase in performance during childhood and adolescence is typically expected, making the logarithmic transformation more plausible in this context, and was expressed as $\theta_{i}=\beta_{0}+\;\beta_{1}{\left(ln[Age]\right)}_{i}+\beta_{2}\;\left[{MPE}_{i}\right]+\;\beta_{3}\;{\left(Sex\right)}_{i}+\beta_{k}\;{Interactions}_{i}+\varepsilon_{i}$. Where residuals $\left(\varepsilon_{i}\right)$ were assumed to follow a normal distribution with a mean of 0 and constant variance $\sigma_{e}^{2}$, that is $\varepsilon_{i}\;\sim \;N(0,\;\sigma_{e}^{2})$. To select the best-fitting model, for both formulations, an exhaustive variable selection approach was implemented, evaluating all possible combinations of the predictor variables ($2^{P}$ models) and selecting the optimal one based on the Mallows’ *Cp* and Bayesian Information Criterion (BIC), selecting the model with the lowest value in each case.

The primary objective of the modeling strategy was predictive rather than purely explanatory. Model parameters were estimated to compute expected scores for new individuals based on demographic variables, thereby forming the foundation for regression-based normative data. To rigorously assess model stability and generalizability, repeated 10-fold cross-validation (CV) was performed, iterated 100 times using 80% (randomly partitioning) of the sample for training. This resampling procedure allowed for the estimation of average out-of-sample prediction error (mean squared error, MSE) across folds, providing a robust internal measure of predictive performance for both the log-transformed and quadratic age models selected according to Cp and BIC criteria.

External validation was further conducted using the independent sample (20%). Each model was fitted on the training data, and its predictive accuracy was quantified on the test data using root mean squared error (RMSE), thereby providing an unbiased estimate of generalization error. The combination of repeated CV and out-of-sample testing enabled a comprehensive evaluation of model performance and supported the reliability of the normative equations when applied to new clinical cases [36]. Finally, assumptions in the final linear regression models were tested. Multicollinearity was evaluated using the Variance Inflation Factor (VIF), with values ≤ 10 considered acceptable. Homoscedasticity was assessed via Levene’s test, and normality of residuals through the Kolmogorov–Smirnov test. To identify potential influential data points, we examined Cook’s distance (Dᵢ), with values exceeding 1 flagged for further inspection [37]. All analyses were conducted in R (version 4.5.1) using the following packages: *lavaan* for structural equation modeling [38], *mIRT* for multidimensional item response theory modeling [39], and *ltm* for latent trait analysis [40].

#### 2.4.3. Normative Data Procedure

Following the procedure described by Rivera et al. (2024) [41], demographically adjusted normative conversions were derived for the estimated ability scores $\left(\hat{\theta }\right)$ from the ROCF copy, ROCF immediate recall, and SVTT $\left(t\in \{{ROCF}_{\mathrm{copy}},{ROCF}_{\mathrm{imm}},\mathrm{SVTT}\}\right)$. These conversions transformed latent trait estimates (*θ*) into percentile ranks that account for individual demographic profiles. First, the expected ability score for each participant $\left({\hat{\theta }}_{it}\right)$ was computed using the regression model coefficients $\left({\hat{\beta }}_{t}\right)$ based on their demographic variables:

$${\hat{\theta }}_{it}\;=\;x_{it}^{⊤}{\hat{\beta }}_{t},$$

where $x_{it}$ represents the individual’s demographic profile. Second, the cumulative probability of the observed ability estimates $\theta_{it}^{\mathrm{obs}}$ for participant i in task t was obtained from the standard normal cumulative distribution function, centered on the expected value $\left({\hat{\theta }}_{it}\right)$ and the model’s residual standard error (${\hat{\sigma }}_{t}$):

$$p_{it}\;=\;Pr\left(Z\leq \theta_{it}^{\mathrm{obs}}\right)=\Phi \left(\frac{\theta_{it}^{\mathrm{obs}}-{\hat{\theta }}_{it}}{{\hat{\sigma }}_{t}}\right),$$

where $\Phi \left(\cdot \right)$ denotes the standard normal cumulative distribution function. Finally, this probability was multiplied by 100 to obtain the corresponding percentile rank $\left({PR}_{it}\right)$:

$${\mathrm{PR}}_{it}\;=\;100\times p_{it}.$$

## 3. Results

### 3.1. Psychometric Properties

For the ROCF copy, CFA supported the assumption of unidimensionality ($x^{2}/df=2.66$; CFI = 0.988; TLI = 0.986; RMSEA = 0.060 [0.053, 0.068]; SRMR = 0.069). Item parameters derived from the IRT analysis (see Table 1) revealed discrimination values ranging from 1.29 (item 16: line) to 3.95 (item 3: diagonal cross). For example, threshold parameters for Item 1 indicate the latent-trait (*θ*) locations at which the probability of scoring at least category *k* (vs. ≤ *k* − 1) equals 50%. Boundary 1 (−2.506) marks *P*(score ≥ 1) = 0.50; boundary 2 (−1.542) marks *P*(score ≥ 2) = 0.50; and boundary 3 (2.292) marks *P*(score ≥ 3) = 0.50.

The DIF analysis identified item 6 due to minor differences in location parameters between boys and girls. These discrepancies reflect minimal variations in the perceived ease of the item across groups; however, the ΔR^2^ of 0.012 indicates that the overall impact on the probability of a correct response is minimal [33]. Therefore, these differences are not substantial enough to affect the interpretation of the subtest, confirming that ROCF copy functions fairly for both sexes (see Table 2).

The measurement precision though TIF for the ROCF copy subtest under the 2PL model exhibits a bimodal pattern, with two distinct peaks of precision across the ability continuum. The highest precision is achieved around *θ* = 0.21, with a SEM of 0.15 indicating excellent reliability (reliability = 0.98) for individuals with higher ability levels. A second, smaller peak appears around θ=0.18, suggesting that the test also provides meaningful information for individuals with lower levels of the latent trait. The instrument demonstrated excellent internal consistency, with an ordinal alpha of 0.94, indicating highly reliable measurement of the underlying construct. The Category Response Curves (CRCs) for all ROCF copy items are presented in Supplementary Material Figure S1.

Regarding the ROCF immediate recall, CFA also supported unidimensionality ($x^{2}/df=2.47$; CFI = 0.978; TLI = 0.975; RMSEA = 0.057 [0.049, 0.065]; SRMR = 0.076). Discrimination parameters ranged from 0.708 (item 15: line) to 1.853 (item 3: diagonal cross). Boundary 1 parameters reflect the level of the latent trait at which there is a 50% chance of obtaining a score of 0.5. The CRCs for items 1 to 6 suggested a limited capacity to differentiate individuals at the highest levels of ability. In contrast, items 8 to 10 required a higher level of proficiency for accurate responses, indicating better performance at the upper end of the trait continuum. For this subtest, items 2 and 4 exhibited small shifts in location parameters between boys and girls, suggesting marginal group-level differences in response probability at certain ability levels. However, the extremely low ΔR^2^ values (0.002 and 0.011, respectively) indicate that the impact of these differences on responses is not significant. This confirms that ROCF immediate recall items can be reliably compared across sexes (see Table 2). The remaining items showed more balanced functioning. The TIF indicated that the instrument achieves its highest precision at the ability level θ=1, with a SEM of 0.315 (reliability of 0.90). These findings suggest that the test is best suited to discriminate among individuals with moderate levels of the latent trait, reaching this range. However, the average information across the ability ranges from −2 to 0 was relatively low (4.20), implying reduced precision for individuals with lower trait levels. Ordinal alpha coefficient of 0.90 reflects a good level of internal consistency for this subtest. Detailed CRCs for all items are available in Supplementary Material Figure S2.

For the SVTT, CFA supported the assumption of unidimensionality (χ^2^/df = 1.32; CFI = 0.959; TLI = 0.956; RMSEA = 0.022 [0.017, 0.027]; SRMR = 0.131). The TIF revealed that the instrument reaches its highest precision at the ability level θ= −2.7, with a SEM of 0.076. These results indicate that the test is optimized to discriminate among individuals with low levels of the latent trait, showing a reliability of 0.99 in this range. However, the information decreases rapidly as ability increases, suggesting lower precision for individuals with average or high levels of the trait. The ordinal alpha of α = 0.90, indicating an adequate level of internal consistency.

The IRT analysis (see Table 3) revealed that item difficulty parameters ranged from −4.799 (item 3: Touch a yellow token) to 0.131 (item 25: Touch the black circle with the red square), indicating that the test is better suited for assessing individuals with lower levels of ability. Discrimination parameters varied widely, from 0.575 (item 25) to 14.664, with the highest values observed for items 5 (Touch a black one), 6 (Touch a green one), and 7 (Touch a white one), suggesting these items are particularly effective at distinguishing between participants across the latent trait continuum. The ICCs for all SVTT items are available in Supplementary Material Figure S3.

In the SVTT subtest, several items showed slight group-related variations in difficulty and discrimination parameters between boys and girls. Nevertheless, the very low ΔR^2^ values indicate that these differences have minimal impact on the probability of a correct response and therefore do not represent meaningful bias. Consequently, the SVTT items can be considered appropriate for comparing cognitive abilities across sexes (see Table 2).

### 3.2. Demographic Variables’ Effect on Neuropsychological Performance

**Figure 1:**
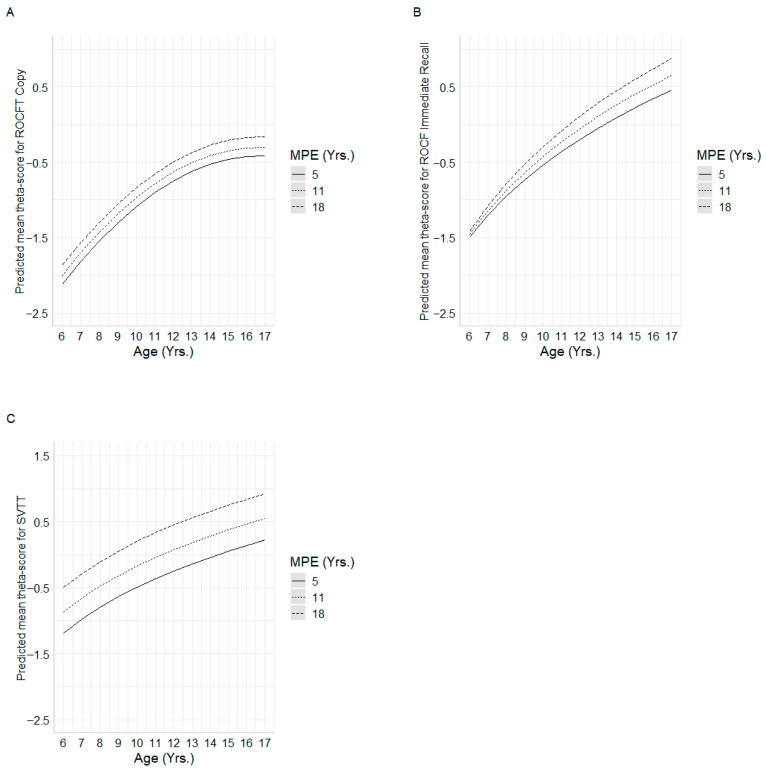
Mean predicted ability scores for each regression model. Subfigures (**A**–**C**) show the interactions between age and MPE for (**A**) = ROCF Copy, (**B**) = ROCF Immediate recall, and (**C**) = SVTT.

For the ROCF copy, four multiple linear regression models based on ability estimates $\left(\theta_{s}\right)$ were compared. Repeated cross-validation revealed that the quadratic age model selected by Mallows’ *Cp* delivered the best performance, with an average of root mean squared error (Mean RMSE) of 0.34 and the lowest MSE in 91% of iterations. In the external test set (20%), differences among models were minimal, with the quadratic *Cp* model again producing the lowest root mean squared error (RMSE = 0.576). Given its superior predictive accuracy and theoretical interpretability, the model included age, age^2^ and MPE as predictors, accounting for 49% of the variance in copy performance (R^2^ = 0.48). The quadratic age predictor showed accelerated gains at younger ages that stabilized in adolescence. In addition, MPE had a positive and significant effect, suggesting that higher parental education years were associated with better performance on the copy task, with this advantage consistently observed across the entire age range (see Table 4, Figure 1A).

Model assumptions were adequately accomplished. No multicollinearity was observed (VIF ≤ 1.001), and no highly influential observations were identified (maximum Cook’s distance = 0.041). Homoscedasticity was confirmed (Levene’s test = 1.9867; *p*-value = 0.116), and residuals did not significantly deviate from normality (*p*-value = 0.732).

Among competing models predicting ROCF immediate-recall *θ*-scores, cross-validation identified the *ln*(age) × MPE interaction model as superior, yielding the lowest mean RMSE (0.630). Model-based trajectories indicated that higher MPE was associated with enhanced recall performance, especially in early childhood, with MPE-related differences converging modestly in late adolescence (see Figure 1B). This specification explained 41.4% of the variance in recall *θ*-scores (R^2^ = 0.414).

For the SVTT, four multiple linear regression models were compared. In the repeated 10-fold, performance was similar across models, with mean RMSE ranging from 0.495 to 0.500. The log-Cp and log-BIC models obtained the lowest RMSE (0.495 and 0.495, respectively), each ranking first in 34% of the iterations, followed closely by the quadratic Cp model (32%). In the external test set, RMSE values were likewise close: 0.715 for both log-Cp and log-BIC, 0.717 for the quadratic Cp model, and 0.720 for the quadratic BIC model. Given its parsimony and robust predictive performance, the final model retained ln(age) and MPE as predictors, jointly accounting for 29% of the variance in SVTT performance (adjusted R^2^ = 0.297). Both *ln*(Age) and MPE exerted significant effects: predicted trajectories demonstrate that higher parental education consistently confers superior SVTT performance across the entire age span, with the greatest MPE-related gains occurring in younger children (see Figure 1C).

Diagnostic tests confirmed that the final model met all assumptions: multicollinearity was negligible (VIF = 1.001), no observations exerted undue influence (maximum Cook’s distance = 0.032), homoscedasticity held (*p*-value = 0.260), and residuals did not depart significantly from normality (*p*-value = 0.180).

### 3.3. Normative Data Application

An online calculator was developed using the Shiny platform to facilitate probability estimation and score interpretation. Clinicians simply need to input the required individual information like namely, test-specific scores, age, MPE, and sex, into the calculator. The tool automatically converts raw item-by-item scores into ability estimates (theta scores), which are then adjusted for sociodemographic variables following the procedure described in Section Normative Data Procedure. As a result, the clinician receives the corresponding percentile for the patient. This tool is freely available to all users at https://elianafuentes.shinyapps.io/itr_col_app/ and will be accessible starting September 2025.

## 4. Discussion

The objectives of this study were to evaluate the validity, reliability, and psychometric properties of both the ROCF and the SVTT using IRT, and to construct normative data for Colombian children, considering the influence of age, educational level, and cultural background. The section is organized according to its aims.

### 4.1. Psychometric Properties

Results supported the unidimensional structure of the ROCF and SVTT, their high reliability and adequate internal consistency. For the SVTT, these findings are in line with previous applications of IRT, such as those reported for the Revised Token Test [42] and the German Token Test [43]. Notably, only Fierro Bósquez et al. [8], working with children from the Waranka Indigenous community, specifically examined the SVTT and also reported evidence of unidimensionality and good reliability.

Regarding the ROCF, in prior research applying the Rasch model, Prieto et al. [44] has provided partial support for its psychometric robustness. However, to our knowledge, the present study is the first to employ the TIF to evaluate the reliability of this instrument, offering a more detailed picture of measurement precision across the ability spectrum.

Regarding the IRT analysis, results for ROCF indicated that all items were able to effectively distinguish between individuals who have adequate visuoconstructional (Copy) and visual memory (Immediate Recall) abilities and those who do not. Additionally, both tasks are suitable for assessing performance across a wide range of ability levels, particularly in those with low levels of skills (e.g., very young children). However, for individuals with high levels of skills, the tasks may not present sufficient difficulty to adequately challenge them. This ceiling effect suggests that, in such cases, the test may be less sensitive to differences between people with higher levels of ability. This is consistent with the observation by [9] that the ROCF copy task is relatively easy. As a result, the measure may not fully capture performance variability among the most capable participants, thereby limiting its diagnostic precision within this subgroup.

Regarding SVTT, the IRT model revealed discrimination parameters within acceptable ranges, indicating an adequate ability to differentiate individuals along the latent trait (auditory language comprehension). However, several items exhibited signs of overfitting or misfit, and should therefore be interpreted with caution, as such deviations may affect the classification accuracy of examinees [45]. A ceiling effect was also observed, reducing the test’s sensitivity to discriminate among high-ability individuals. This pattern is consistent with the TIF results, which indicate that the instrument provides its highest measurement precision at lower ability levels. Similar findings were reported by [8,14], where no significant performance gains were observed in children aged 14 and 11, respectively, further supporting the presence of a ceiling effect. This does not indicate a flaw in the instrument, as it aligns with its original purpose of detecting comprehension deficits in individuals with aphasia. Accordingly, the items were calibrated so that individuals without language disorders would solve them with high probability, thereby reflecting the test’s clinical design.

In contrast, several items displayed optimal psychometric properties and contributed meaningful information across the full range of ability. These results suggest that, although the SVTT performs adequately, item-level refinements, such as removing or replacing overly easy items, could enhance measurement precision and reduce model distortion. Nonetheless, such refinements should be considered with caution, as modifications aimed at improving psychometric properties in healthy samples may not necessarily align with the test’s original clinical purpose.

The DIF analysis conducted revealed that none of the items in the ROCF-Copy, ROCF-Immediate recall, and SVTT subtests exhibited significant sex-related effects. These findings are consistent with previous research showing that, even when DIF is detected, the effects are often clinically irrelevant when ΔR^2^ values are small [46]. Therefore, instruments can be considered equitable and appropriate for comparing cognitive abilities between boys and girls.

### 4.2. Demographic Variables and Normative Data

Regarding the influence of sociodemographic variables on test performance, the most robust predictors were age^2^, *ln*(age) and, in some cases, MPE and its interaction with age. The use of different age transformations reflects the nonlinear nature of cognitive development: age^2^ captures the rapid gains observed in early childhood followed by a plateau in adolescence, whereas *ln*(age) better models the decelerating growth rate across the developmental span. These patterns highlight the complexity of modeling developmental trajectories, where cognitive growth is substantial at younger ages but tends to slow down as children approach adolescence [3].

Parental education emerged as a key variable, highlighting the important role of the family’s educational environment in shaping cognitive performance. Children whose parents had more years of formal schooling consistently outperformed their peers, in line with findings from previous Latin American pediatric samples [8,14,34]. Beyond a main effect, the interaction between age and MPE suggests that parental education may modulate the pace of cognitive growth; it means that in some cases accelerating developmental gains, while in others buffering against a slower trajectory. This interaction underscores the broader impact of the sociocultural environment on child cognition, particularly in domains central to language [47], visuoconstructive abilities [48], and visual memory [49]. This pattern is consistent with evidence that parental education often reflects broader socioeconomic advantages, including access to adequate nutrition and healthcare (Ross & Mirowsky, 2011) [50], higher-quality educational opportunities (e.g., access to material and educational resources), and cognitively enriched home environments (e.g., shared reading, elaborated conversations, exposure to complex vocabulary, offer opportunities for bilingual or second-language experiences [51]). Indeed, previous evidence shows that parental education may operate indirectly through the quality of the home environment and the development of children’s self-regulatory and attentional capacities [52] as well as through access to cognitively enriching school contexts and family learning practices that mediate socioeconomic effects on cognitive and language outcomes [53].

These findings emphasize that performance on the SVTT and ROCF should be interpreted in the context of parental education and its broader socioeconomic correlates discussed above, especially in Latin American settings where structural inequalities in access to quality education persist [54]. Without such contextualization, children from less advantaged educational environments may be at risk of misinterpretation, with typical variability being mistaken for cognitive impairment. This perspective aligns with calls to interpret neuropsychological results within their ecological and social context to avoid pathologizing expected variability.

Also, these sociodemographic influences highlight the need for precise methods of capturing individual differences. In this regard, a key contribution of this study is the establishment of normative data based on ability scores derived from IRT, rather than traditional total scores. While prior studies relied primarily on raw totals, the use of ability scores provides a more precise and psychometrically grounded representation of performance, offering finer discrimination across different ability levels [55]. This approach addresses some of the limitations of ceiling effects and enhances the interpretability of individual differences across the developmental spectrum.

Finally, the exhaustive selection of predictor variables combined with repeated cross-validation strengthens the robustness and generalizability of the models. By minimizing overfitting and ensuring stable parameter estimates, this methodology provides a more reliable framework for understanding the determinants of cognitive performance and for developing normative references that can inform both clinical practice and research [56].

The calculator developed in this study is intended as a supportive tool to facilitate the interpretation of test scores, particularly in settings where rapid or standardized evaluation is needed. Importantly, it is not designed to replace professional clinical judgment. Clinicians should integrate its outputs with their broader assessment of the child, considering contextual factors such as educational environment, language exposure, and socioeconomic background, as well as qualitative observations from testing.

### 4.3. Limitations, Strengths, and Clinical Implications

The lack of appropriate normative data has tangible implications. Clinicians serving underrepresented populations may not have reliable normative references to interpret performance accurately. In such cases, professionals might inadvertently pathologize normal variability or, conversely, fail to detect true cognitive difficulties. In the absence of appropriate data, such as those provided in this study, there is a risk of relying on norms that are not representative of the populations they serve, which may lead to diagnostic inaccuracies and inequitable clinical decisions.

This study has some limitations that should be considered. First, only variables traditionally identified in the literature as relevant were examined. For future research, it will be important to consider valuable external predictors or moderators of cognitive and behavioral performance such as socioeconomic status, screen exposure or nutrition. Socioeconomic status may be operationalized through parental education, household income, occupation, or composite indices. Screen exposure can be measured in terms of duration (hours per day), type (educational vs. recreational), and timing (e.g., before or after bedtime). Nutritional status can be assessed using indicators, such as Body Mass Index (BMI) normalized for age and sex (WHO BMI-for-age percentiles or z-scores), low height-for-age (WHO z-scores), low weight-for-height (WHO z-scores), or micronutrient deficiencies.

Although the empirical superiority of IRT-based scoring has been demonstrated in terms of lower Type I error, greater statistical power, and more accurate effect-size estimation, future external validation should be conducted to establish its predictive utility using independent criteria such as teacher or parent ratings, academic performance, and daily functioning measures. Moreover, metrics like Area Under Curve for discrimination, and Root Mean Square Error and Cross-validation systems for predictions should be implemented.

Also, even though the sample was demographically diverse and the IRT framework minimizes sample dependence in parameter estimation, it did not include children from rural or indigenous communities. This limitation constrains the generalizability of the findings to the broader Colombian population. Moreover, bilingual status was not systematically characterized, which may be relevant given that language exposure and use can influence cognitive and linguistic performance. However, bilingualism is relatively uncommon in the urban regions represented in this study and is more prevalent in indigenous populations that were not included in the sample.

Finally, while regression-based norms offer greater flexibility and precision, they assume that the relationship between predictors and test performance remains stable across the entire ability spectrum. In other words, the effect of a predictor on performance is assumed to be the same for both low-ability and high-ability individuals. But, from a clinical perspective, these limitations underscore the importance of critically examining the impact of cultural, linguistic, and socioeconomic diversity when interpreting test results. This reflective stance is particularly relevant in Latin American contexts, where variability in educational quality, language exposure, and access to resources can substantially shape cognitive performance. Such sources of heterogeneity, together with potential ceiling effects in higher-ability groups, may reduce the sensitivity of the models to detect meaningful differences. When test scores approach ceiling levels, clinicians are advised to incorporate complementary and more demanding measures, such as design fluency tasks, delayed recall, or complex language assessments. At the same time, the presence of ceiling effects provides valuable insight for future test development: refining or adding items that target higher-order cognitive abilities could enhance the discriminatory power of the instruments for high-performing children.

Despite this, this study offers several important strengths. First, the development of regression-based normative data, accompanied by an automated online calculator, significantly improves the clinical utility of the ROCF and SVTT. This tool allows professionals to derive demographically adjusted ability estimates (*θ*-scores) and percentiles with greater diagnostic precision, surpassing traditional norm table approaches.

Second, the application of IRT, still underutilized in Latin American pediatric contexts, offers substantial methodological advantages. IRT allows finer-grained estimation of individual ability, identifies items with poor psychometric performance (misfit, ceiling effects), and provides the foundation for future development of culturally adapted, shortened, or computerized versions of the tests [57].

Third, the study focuses on a large, diverse sample of Colombian children and adolescents, representing multiple cities and sociodemographic backgrounds. Given the scarcity of regionally specific data in Latin American populations, particularly among children, this sample adds substantial value and addresses gaps noted in international reviews [5]. Most IRT-based neuropsychological studies to date have focused on adult samples or clinical populations, making this study particularly valuable due to its focus on a pediatric population and its broader geographical scope.

The IRT framework allows the examination of group differences in item parameters (e.g., children with low vs. high SES). Future studies could incorporate SES, screen exposure, and nutrition into IRT models in several ways: (1) As grouping variables in DIF analyses, for example, using Multiple-Group IRT to determine whether items function differently across low- and high-SES groups; however, there is a risk of losing information while dichotomizing the variables. (2) As covariates in explanatory IRT models (regression within IRT), allowing SES, screen exposure, and nutrition to predict the latent trait (*θ*) and modeling children’s probability of correct or endorsed responses as a function of both their trait level and these covariates.

## 5. Conclusions

This study offers a robust psychometric and normative foundation for clinicians working with Colombian Spanish-speaking children and adolescents. The integration of IRT, regression-based normative modeling, and user-friendly digital tools represents a meaningful advancement in neuropsychological assessment, particularly in culturally and socioeconomically diverse settings. Together, these innovations provide health professionals with a highly accurate and accessible resource for evaluating visual–spatial construction, immediate visual memory and verbal comprehension functioning in pediatric populations.

## Figures and Tables

**Table 1:** ROCF item parameters, based on item response theory.

Items	ROCF-Copy				ROCF-Immediate Recall			
	Boundary 1	Boundary 2	Boundary 3	*a*	Boundary 1	Boundary 2	Boundary 3	*a*
Item 1	−2.506	−1.542	2.292	1.824	−1.89	1.059	6.951	0.934
Item 2	−2.428	−1.186	2.262	2.547	−1.759	−0.395	6.382	1.717
Item 3	−1.994	−1.2	1.431	3.958	−0.416	0.434	4.11	1.853
Item 4	−2.25	−1.389	1.583	3.125	−1.023	−0.443	5.823	1.833
Item 5	−2.666	−1.605	2.155	2.241	−0.953	−0.378	5.592	1.661
Item 6	−2.746	−1.121	2.496	2.125	−0.531	1.128	4.842	1.381
Item 7	−1.789	−1.412	1.869	2.146	0.637	0.951	4.397	1.621
Item 8	−2.355	−0.972	1.91	2.832	−0.451	0.781	4.033	1.833
Item 9	−2.355	−1.424	1.791	2.726	−0.033	1.051	6.667	0.965
Item 10	−2.44	−1.595	2.096	2.002	2.139	2.804	6.293	0.887
Item 11	−2.868	−1.579	2.032	1.913	−1.4	0.15	4.976	1.413
Item 12	−2.356	−1.415	1.773	2.705	0.385	1.292	6.168	1.038
Item 13	−2.882	−1.543	2.855	1.837	−1.898	−0.633	10.005	1.214
Item 14	−2.917	−1.434	2.552	1.592	−1.514	0.342	8.378	0.777
Item 15	−2.744	−1.595	2.723	1.516	0.124	0.991	8.911	0.708
Item 16	−3.064	−2.059	7.59	1.293	−0.734	−0.249	8.532	0.852
Item 17	−2.455	−1.323	1.937	2.6	−1.456	1.252	5.609	1.145
Item 18	−3.009	−1.387	2.604	1.587	−1.282	1.785	7.766	0.779

Note. *a* = Item discrimination parameter. Boundary 1 = response category 0.5; Boundary 2 = response category 1; Boundary 3 = response category 2. Elements for each item: 1 = cross; 2 = large rectangle; 3 = diagonal cross; 4 = horizontal line; 5 = vertical line; 6 = small rectangle; 7 = small segment; 8 = parallel lines; 9 = triangle; 10 = line; 11 = circle with 3 dots; 12 = parallel lines; 13 = triangle; 14 = diamond; 15 = line; 16 = line; 17 = cross; 18 = square.

**Table 2:** Differential item functioning for ROCF and SVTT.

		Boys					Girls			
Subtest	Item	ΔR^2^	C1	C2	C3	C4	C1	C2	C3	C4
ROCF copy	6	0.012	2.16	−2.28	−1.59	−0.09	1.52	−3.28	−2.59	−0.28
ROCF Immediate Recall	2	0.002	1.64	−2.18	−1.53	−0.15	2.61	−1.52	−0.94	−0.03
	4	0.011	2.3	−1.04	−0.69	−0.26	2.33	−0.81	−0.58	0.02
**Subtest**	**Item**	**ΔR^2^**		***b***	***a***			***b***	***a***	
SVTT	2	0.002		14.08	−2.55			1.03	−6.06	
	4	<0.001		14.08	−2.55			0.2	−29.12	
	8	0.046		0.92	−4.12			4	−2.38	
	15	0.042		2.02	−1.61			1.44	−2.25	
	16	0.007		1.01	−1.8			1.91	−0.99	

Note. C1 = Response category 2, C2 = Response category 1, C3 = Response category 0, *b* = Item difficulty parameter, *a* = Item discrimination parameter; ROCF = Rey–Osterrieth Complex Figure.

**Table 3:** Shortened Version of the Token Test item parameters, based on item response theory.

	*a*	*b*		*a*	*b*
Item 1	2.130	−3.081	Item 19	1.656	−1.918
Item 2	3.027	−3.094	Item 20	1.419	−1.286
Item 3	1.094	−4.799	Item 21	1.375	−1.782
Item 4	2.405	−3.41	Item 22	1.435	−2.303
Item 5	14.644	−2.715	Item 23	1.455	−2.005
Item 6	14.644	−2.715	Item 24	1.134	−2.585
Item 7	14.644	−2.715	Item 25	0.575	0.131
Item 8	1.778	−3.404	Item 26	0.829	−2.049
Item 9	3.435	−3.196	Item 27	1.43	−1.39
Item 10	1.297	−3.361	Item 28	0.917	−2.242
Item 11	1.952	−3.326	Item 29	0.911	−1.503
Item 12	1.296	−3.468	Item 30	1.288	−1.655
Item 13	1.266	−3.584	Item 31	0.765	−2.764
Item 14	1.405	−3.39	Item 32	1.288	−1.193
Item 15	1.996	−2.566	Item 33	0.918	−3.312
Item 16	1.039	−2.864	Item 34	0.662	−3.207
Item 17	1.388	−2.033	Item 35	0.833	−3.143
Item 18	1.823	−2.423	Item 36	1.144	−0.330

Note: *a* = Item discrimination parameter. *b* = Item difficulty parameter. Parameters estimated using a 2-parameter logistic IRT model. Brief labels for each item: 1 = Circle; 2 = Square; 3 = Yellow piece; 4 = Red piece; 5 = Black piece; 6 = Green piece; 7 = White piece; 8 = Yellow square; 9 = Black circle; 10 = Green circle; 11 = White square; 12 = Small white circle; 13 = Large yellow square; 14 = Large green square; 15 = Small black circle; 16 = Red circle + green square; 17 = Yellow + black squares; 18 = White square + green circle; 19 = White + red circles; 20 = Large white circle + small green square; 21 = Small black circle + large yellow square; 22 = Large green + red squares; 23 = Large white square + small green circle; 24 = Red circle on green square; 25 = Black circle with red square; 26 = Black circle and red square; 27 = Black circle or red square; 28 = Green square far from yellow square; 29 = If blue circle, red square; 30 = Green square next to red circle; 31 = Squares slowly, circles quickly; 32 = Red circle between yellow and green squares; 33 = All circles except green; 34 = Red circle; no, white square; 35 = Instead of white square, yellow circle; 36 = In addition to yellow circle, black circle.

**Table 4:** Final multiple linear regression models.

Score	Predictor	B	Std. Error	t	Sig.	Adjusted R^2^	$s_{\varepsilon }$
ROCF-copy	Intercept	−1.153	0.104	−11.128	<2 × 10^−16^	0.4861	0.586
	Age	10.398	0.586	17.736	<2 × 10^−16^		
	Age^2^	−2.912	0.587	−4.964	1.07 × 10^−6^		
	MPE	0.019	0.008	2.361	0.018		
ROCF-immediate recall	Intercept	−4.640	1.023	−4.538	7.77 × 10^−6^	0.414	0.790
	*ln*(Age)	1.742	0.425	4.093	5.25 × 10^−5^		
	MPE	−0.040	0.080	−0.494	0.622		
	*ln*(Age) × MPE	0.025	0.033	0.759	0.448		
SVTT	Intercept	−3.870	0.258	−15.004	<2 × 10^−16^	0.2971	0.702
	*ln*(Age)	1.352	0.098	13.826	<2 × 10^−16^		
	MPE	0.053	0.008	6.467	2.26 × 10^−10^		

Note. ROCF = Rey Osterrieth Complex Figure Test; MPE = Mean Parent years of Education; SVTT = Shortened Version of the Token Test; B = Beta; $s_{\varepsilon }$ = Residual Standard deviation.

## Data Availability

The data and code can be provided upon request.

## Supplementary Materials

The following supporting information can be downloaded at https://www.mdpi.com/article/10.3390/healthcare13212683/s1. Figure S1 presents the category response curves for the ROCF-Copy task (Line 1 = response category 2; Line 2 = response category 1; Line 3 = response category 0.5; Line 4 = response category 0). Figure S2 shows the category response curves for the ROCF-Immediate Recall task (Line 1 = response category 2; Line 2 = response category 1; Line 3 = response category 0.5; Line 4 = response category 0). Figure S3 displays the item characteristic curves for the Shortened Version Token Test.
