# Supplementary material for: Advancing Pediatric Cognitive Health: Psychometric Evaluation and IRT- and Regression-Based Norms for Two Neuropsychological Measures in Colombian Children and Adolescents
Source: Healthcare (Basel). 2025 Oct 23;13(21):2683. doi: 10.3390/healthcare13212683 (PMC12610619; doi:10.3390/healthcare13212683)

**Figure S1.** ROCF-Copy Category response curves

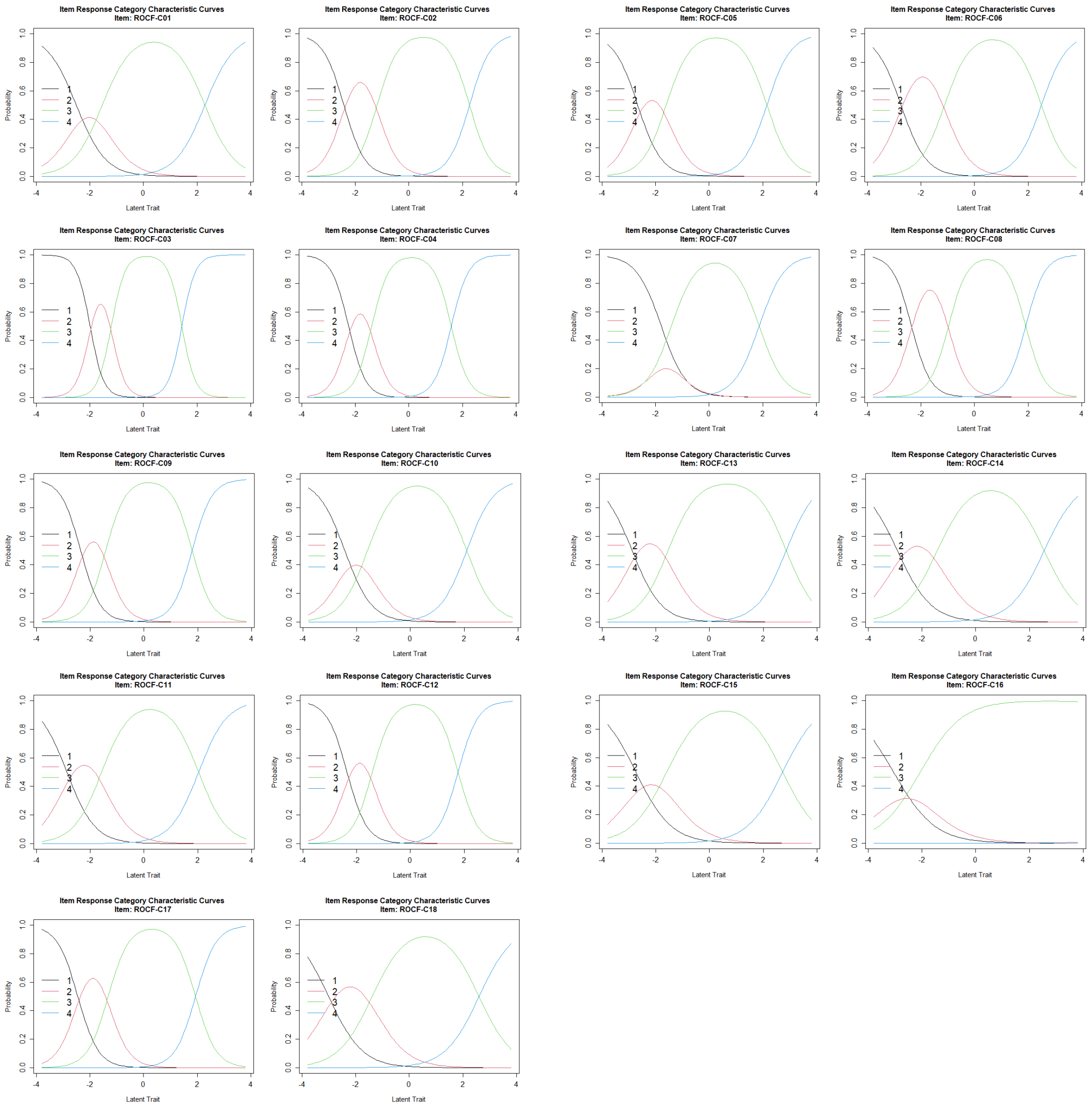


*Note*. Line 1= response category 2; Line 2= response category 1; line 3= response category = 0.5; line 4= response category = 0

**Figure S2.** ROCF-Immediate Recall Category response curves

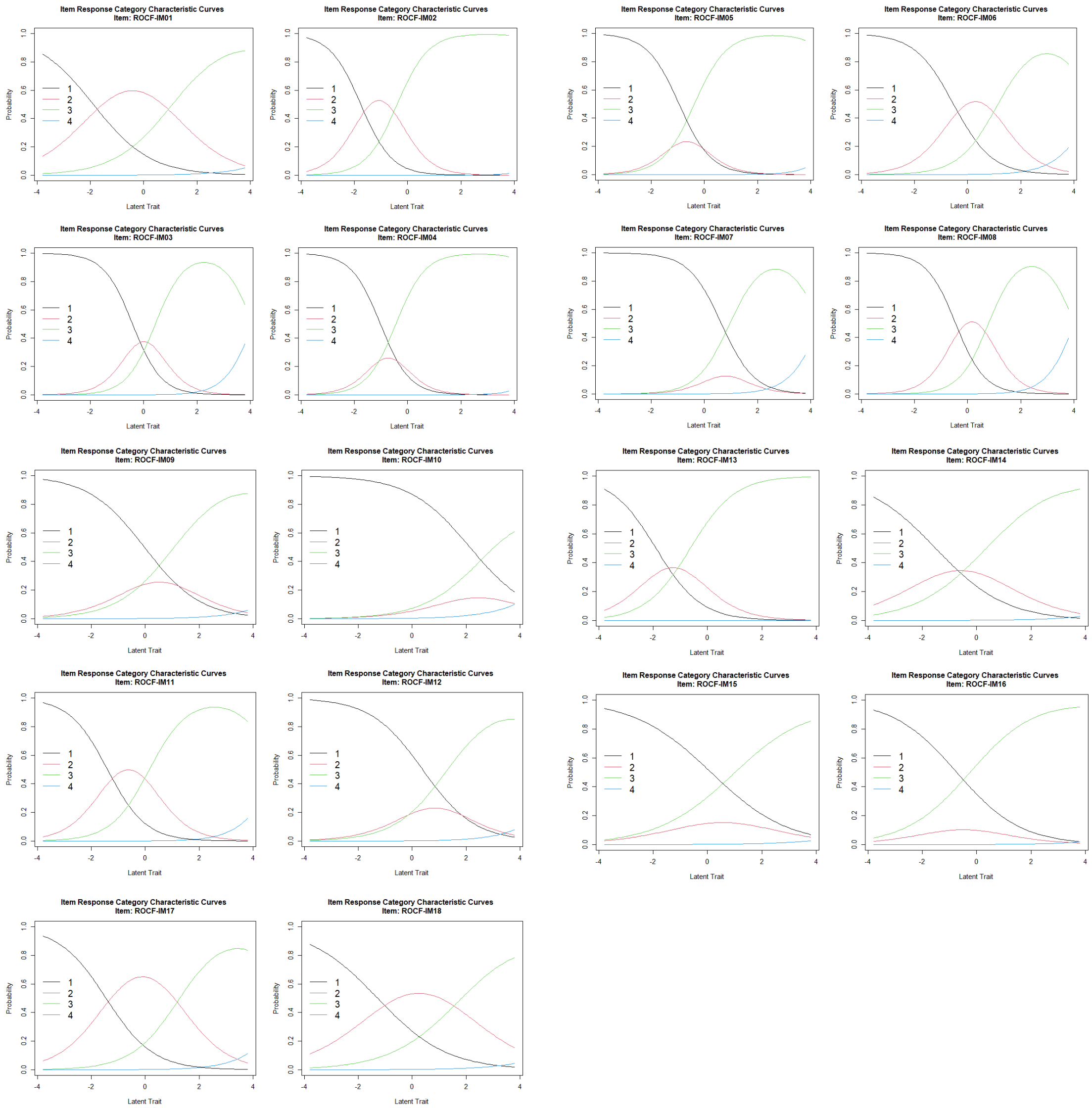


*Note*. Line 1= response category 2; Line 2= response category 1; line 3= response category = 0.5; line 4= response category = 0

**Figure S3**. Shortened Version Token Test Item Characteristic Curves

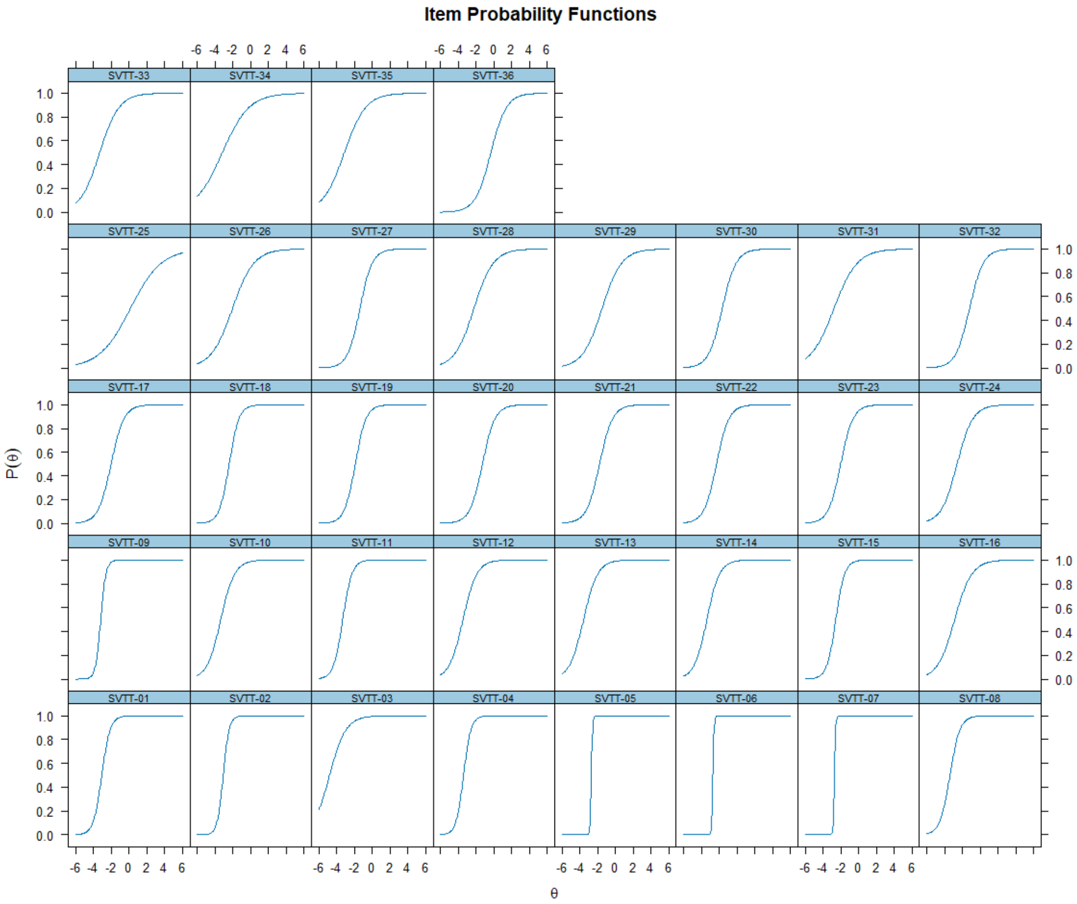

Supplement: Supplementary file 1 [file healthcare-13-02683-s001.zip › healthcare-3896046-supplementary.pdf]
